# Global Insights on the Involvement of Ethnic Minority Populations in Health and Social Care Research Priority Setting: A Systematic Scoping Review

**DOI:** 10.1007/s40615-025-02377-x

**Published:** 2025-03-13

**Authors:** Winifred Ekezie, Shabana Cassambai, Ffion Curtis, Barbara Czyznikowska, Lauren L. O’Mahoney, Andrew Willis, Shavez Jeffers, Ruksar Abdala, Ayesha Butt, Yogini Chudasama, Kamlesh Khunti, Azhar Farooqi

**Affiliations:** 1 National Institute for Health and Social Care Research (NIHR), Applied Research Collaboration East Midlands (ARC EM), Leicester, UK; 2 Diabetes Research Centre, University of Leicester, Leicester, UK; 3 Centre for Ethnic Health Research, University of Leicester, Leicester, UK; 4 Centre for Health and Society, Aston University, Birmingham, UK; 5 Liverpool Reviews and Implementation Group (LRiG), Institute of Population Health, University of Liverpool, Liverpool, UK; 6 HRB Clinical Research Facility & School of Public Health, University College Cork, Cork, Ireland; 7 NIHR Biomedical Research Centre (BRC), Department of Respiratory Sciences, University of Leicester, Leicester, UK; 8 Leicester Real World Evidence, University of Leicester, Leicester, UK; 9 East Midlands Regional Research Delivery Network (EM RRDN) NIHR, Leicester, UK

**Keywords:** Scoping review, Ethnicity and race, Ethnic minorities, Health research, Research priorities, Social care

## Abstract

**Background:**

Representing all population groups in health and social care research is essential for generating research relevant to decision making in everyday clinical and social healthcare policy and practice. Conducting research that is relevant to all, starts with ensuring equitable representation in research priority selection. This scoping review aimed to identify evidence of published and good practices in health and social care research priority-setting activities, which included people from ethnic minority backgrounds.

**Methods:**

The search was conducted using MEDLINE, CINAHL, Cochrane Library, PsycINFO, and Scopus databases, following the Preferred Reporting Items for Systematic Reviews and Meta-Analyses extension for Scoping Reviews (PRISMA-ScR) guideline. Studies that reported including ethnic minority community members in health and social care research priority setting from 2010 were considered. The research priority processes were evaluated using a checklist of good practices in research priority settings.

**Findings:**

Forty-seven articles representing 12 countries and various health topics were included. Group discussion was the most common approach for conducting the research priority setting activities. No study addressed all 20 recommended research priority–setting good practice principles. Most studies provided sufficient information about the context of the priority-setting exercise. Examples of good practices included community advisory boards, local approaches to health research, and multi-disciplinary steering groups.

**Conclusion:**

Representation of ethnic minority populations’ involvement in research across different countries and broader health and social care areas is limited. Recommendations to address these challenges are presented and could help inform researchers, funders, and policymakers to understand what health and social care research topics are prioritised by ethnic minority communities.

**Supplementary Information:**

The online version contains supplementary material available at 10.1007/s40615-025-02377-x.

## Introduction

Health care and clinical decision-making need to be informed by research that takes into account the social determinants of health, which are known to impact disease incidence and treatment outcomes [1]. However, ethnic minority populations, defined as “groups within a country or community which has different national or cultural traditions from the larger, dominant population” [2], are often under-represented in research studies that inform decision making. This poor representation compromises the external validity of research, thus making research findings less generalisable and relevant for some ethnic groups [1, 3–6].

Various types of research are conducted to improve health and social care, including clinical, biomedical, health services, and public health research. Findings from these various types of research can lead to changes in treatments, policies, and care that affect all population groups. As such, increased ethnic minority population research participation is crucial for understanding the aetiology and management of health conditions, reducing health inequity and encouraging culturally competent health care [7–9]. However, evidence-based strategies to encourage recruitment from these groups are lacking [10], and identified barriers to participation for those from ethnic minority groups include poor understanding of their interests and needs and a lack of additional resources to support and sustain trusted relationships [1, 10]. These observations are partly because effective solutions are often context (population, health condition, or setting) specific, making it challenging to decipher steps taken in research development processes. As a result, the priorities of ethnic minority communities are often not appropriately considered. Also, having diverse representation in research is essential for promoting equitable research and reducing health outcome disparities [11]. Hence, more effort is required to proactively encourage and implement relevant research among less-engaged groups.

Clinical randomised trials with “pragmatic intent” are designed to be most relevant in informing healthcare treatment decisions [12]. Trials designed to fit this purpose should aim to include a participant group that mirrors the group of patients who would receive the new treatment if it were delivered in routine care. Such trials aim to select a primary outcome most relevant to those making healthcare decisions, including patients themselves. Consequently, designing efficient trials requires equitable representation in priority and outcome selection. Thus, research priority setting assists researchers and policymakers in addressing areas of greatest need, and best practice entails identifying and prioritising research important to stakeholders [13], including patients and carers providing direct experience from health conditions for the research context. However, published research on priority setting among ethnic minorities lacks transparency in the research process, and most do not report the involvement of ethnic minorities in developing the research agenda [1, 14, 15].

This scoping review, therefore, aimed to identify and collate all available evidence of research priority settings conducted with ethnic minority communities globally. The review explored research priority settings and good practices among ethnic minorities, which would help ensure that researchers and those who fund health research know what matters to ethnic minority communities, including patients, carers, and clinicians. The review research questions were as follows: Where have ethnic minority populations been involved in health and social care priority research settings? What were the research priorities as identified by the ethnic minority populations? What were their experiences and perceptions of involvement in research priority-setting exercises?

The scoping review is part of the National Institute for Health and Care Research, Applied Research Collaboration (NIHR ARC) REPRESENT study on health and social care research priority setting among ethnic minority populations [16] and will contribute to shaping future research that aims to improve the health outcomes of ethnic minorities as well as increase their participation in research.

## Methods

The JBI systematic/scoping review guidelines and the Arksey and O’Malley methodology framework were used to inform the development of this scoping review protocol, written in accordance with the PRISMA extension for scoping reviews (PRISMA-ScR) checklist [17, 18]. The protocol for the scoping review was registered on Open Science Framework (OSF) registries [19].

Inclusion criteria were based on participants (ethnic minority populations), concept (research priority set by ethnic minority populations), and context (health and social care research studies). Ethnic minorities were defined as “a group within a country or community which has different national or cultural traditions from the larger, dominant population” [2]. Specific inclusion criteria were developed for quantitative, qualitative and mixed methods studies.

### Study Search and Selection

We searched the following databases: MEDLINE (PubMed), CINAHL, Cochrane Library, PsycINFO, Scopus Cochrane Library, and PROSPERO. The search was undertaken in January 2022, with additional searches performed in June 2023. Key search terms included ethnic minorities, priority setting, health concerns, and health research planning. Studies were included if they focused on obtaining research priorities for ethnic minority populations, including migrants, indigenous, and aboriginal communities. The search from OVID MEDLINE is shown in Supplementary 1. Database searches were supplemented with internet searches (i.e., Google Scholar) with forward and backward citation tracking from included studies and related review articles. Studies were included if they reported quantitative and/or qualitative data. A similar review was conducted from database inception in 2020, but this study included community, health providers, and expert ethnic minorities [14]. Our study aligns with that review, but we focus only on the involvement of ethnic minority community members. Hence, we considered only studies published from the year 2010 and in the English language.

References from the database searches were imported to Rayyan review manager software [20]. Duplicates were removed, and titles and abstracts were reviewed independently by two reviewers (WE, SC, FC, AW, LLO, RA, and AB). The full-text screening was also performed in duplicate by two reviewers (WE and SC), and conflicts were resolved by discussion. Whenever consensus could not be reached, a third reviewer (FC) was involved. The reference lists of the included studies were reviewed for additional relevant articles. Studies were screened against the inclusion and exclusion criteria presented.

#### Inclusion Criteria

- ***Participants***: Ethnic or racial minority community members
- ***Concept***: Research priority set by ethnic minority populations
- ***Context***: All health and social care research studies
- ***Types of study***: All primary research study designs published in any country
- ***Time span***: Published online from January 2010
- ***Language***: English

### Charting the Data

A data extraction form was developed and piloted in Microsoft Excel to extract all relevant data required for this review from the included studies. Data from all included studies were extracted and variables extracted included author(s), year, country, health topic/scope, study population, study design, methods of recruitment and data collection, research settings and questions, theoretical framework used and experience and challenges of the priority-setting activities. One reviewer performed the extraction, and another checked it for accuracy. Data analysis was performed by WE using descriptive synthesis to summarise the study characteristics and research priority trends.

### Quality Appraisal

No standard study quality assessment was performed, as this is not a requirement of scoping reviews [18]. However, a methodological framework for evaluating research priority processes was used to assess each included study [21] (Supplementary 2). This framework checks nine common areas of health research priority setting: context, comprehensiveness, inclusiveness, information gathering, implementation planning, focus criteria, decision-making methods, evaluation, and transparency. The nine areas are further split into 20 good practice criteria guidance, which can be used to design a structured priority setting process for patients and the public alongside healthcare providers and researchers. This framework is one of a few globally accepted and standardised guidance for health research priority setting, which researchers and policymakers can select from [22, 23]. The framework has also been used extensively for various populations for health research, practice, and policy development [24, 25], but there is no evidence of its use for engaging ethnic minority groups. A similar review to evaluate research priority–setting exercises and identify issues in ethnic minority groups has used the same framework [14]. That review assessed the involvement of both ethnic minority communities and health service providers and experts. Our review builds on that but focuses only on the community members.

After appraising the included studies against the 20 checklist items across the nine areas in the framework, each study was scored by the proportion of items identified within the research priority setting exercises reported. However, the quality of the studies was not used to determine inclusion in the review; hence, there was no cut-off point, and all studies matching the inclusion criteria were included in the studies regardless of the quality ratings.

## Results

**Fig. 1:**
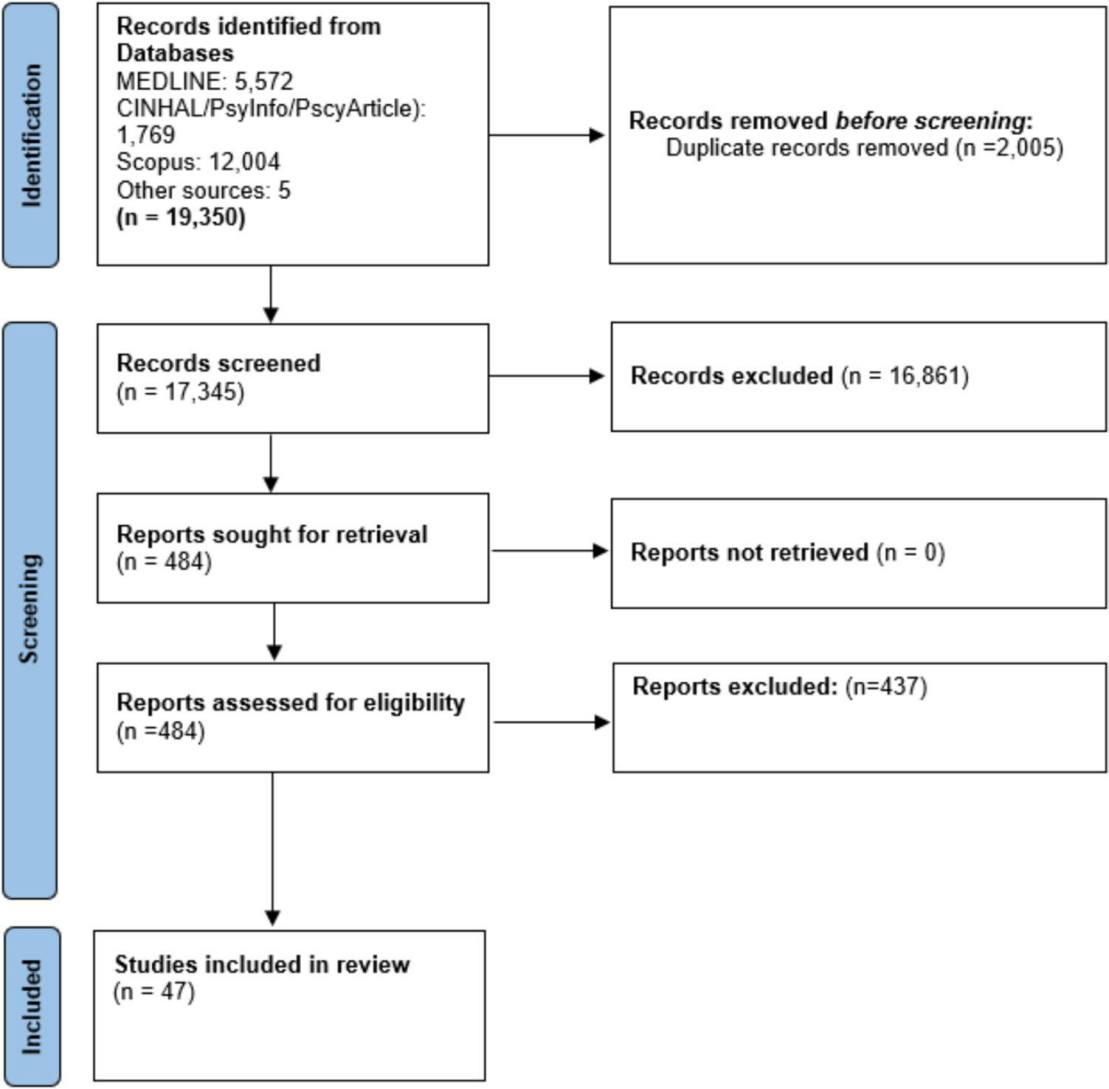
Flow diagram of included study selection

After duplicate removal, searches yielded a total of 17,587 unique records (Fig. 1). Of these, we considered 479 records for full-text screening against the eligibility criteria. A total of 432 studies that had no clear information on the involvement and contribution of ethnic minority communities in the priority setting exercises were excluded. Finally, we included 47 articles in this review. A full list of articles and a description of their main characteristics is provided in Supplementary Table 1.

### Descriptive Analysis of Research Trends on the Priority Setting Involving Ethnic Minority Groups

The 47 included studies spanned from 2010 to 2023, with a pronounced increase in the number of articles published by year from 2017. The studies spanned 12 countries, with the most published in the USA (*n* = 22, 47%), UK (*n* = 8, 17%), and Australia (*n* = 7, 15%). Other countries were Spain (*n* = 2), Germany, India, Luxemburg, Myanmar, New Zealand, Saudi Arabia, and South Africa, with one included study each.

The included articles represented a range of health areas but mostly related to general health issues (*n* = 9, 21%), mental health (*n* = 7, 16%), cancer (*n* = 5, 12%), and diabetes (*n* = 4, 9%). Topics identified in two studies were obesity, HIV, healthcare access, and refugee health. Other issues explored in only one study included those focused on a health condition (knee replacement surgery, chronic pain, maternal health, overactive bladder, and Parkinson’s), social-related issues (sexual violence), and methodological issues (primary care and biomedical research). For the study design approach adopted for collecting information, most used more than one approach, with group discussion being the most common (*n* = 20, 45%), followed by surveys, including Delphi studies (*n* = 18, 41%), and key informant interviews (*n* = 11, 25%). Other methods included participatory action research, community mapping, photovoice, and storytelling.

Based on how participants were reported in the included studies, the population groups were grouped into three categories: ethnic minorities (*n* = 35, 80%), migrants (*n* = 8, 18%), and underserved (*n* = 5, 11%). Ethnic minorities included indigenous populations and minority ethnicities in different countries. Migrants included those born outside the country of study, including those granted refugee status and those in the process of seeking asylum, while the underserved groups included medically underrepresented communities, including people under 18 years old and hard-to-reach communities. In addition, some studies also included non-community individuals and organisations in the priority exercise, such as health practitioners (*n* = 16, 36%), academic researchers (*n* = 11, 25%), and government policymakers (*n* = 2, 5%).

### Study Quality Assessment for Good Practice in Research Priority Setting

A summary of the assessment of included studies using the “A checklist for health research priority setting: nine common themes of good practice” is presented below (see Supplementary 4).

#### Theme 1: Context

Every study reported some contextual factors, with studies reporting on at least four of the seven factors. All studies presented a clear focus of the priority-setting exercises; 94% (*n* = 44) reported the values and principles guiding the activities, and 57% (*n* = 27) included information regarding the resources used. For the environmental contexts, 51% (*n* = 24) described the health environment, 95% (*n* = 45) reported the research environment, 19% (*n* = 9) described the political environment, and 9% (*n* = 4) discussed the economic situation.

#### Theme 2: Use of a Comprehensive Approach

All 47 included studies reported using a framework or guideline to conduct their research. However, five studies used priority-setting exercise frameworks; most used the James Lind Alliance (JLA) approach [24–27]. One study proposed a new method for initiating patient and public involvement, the Research Prioritization by Affected Communities (RPAC) framework [28]. The other studies used conceptual and methodological frameworks such as community-based participatory research (CBPR) [29–34], socio-ecological framework [35, 36], Consolidation Framework for Implementation Research (CFIR) [37], national frameworks [38–40], and other adapted approaches [41] to guide their research priority setting exercises.

#### Theme 3: Inclusiveness

Thirty-six studies (77%) described the participants involved in the process, and nine studies did not provide any demographic details. This included demographic information about their age, gender/sex, education, ethnicity, employment, expertise, income, disability, and language proficiencies. The report of these information varied across all 36 studies. Of the 28 studies reporting gender representation, the majority (*n* = 23, 82%) recruited a higher proportion of females. Information about the education level and expertise of the participants was reported in 11 studies, respectively. Participants also included non-community members such as service providers, academics, researchers, and practitioners. Regional participation representation in national and global level priority–setting processes was reported in 23 studies (49%), while representation from health sectors and other constituencies, including social care, was reflected in 30 studies (64%).

#### Theme 4: Information Gathering

Most studies (*n* = 24, 51%) reported on the information and sources used and involved minority communities in planning priority setting activities. This often included partnership or consultation with ethnic minority community representatives during study design [30, 33, 42–46]. In such studies, some conducted community mapping, and several established a community advisory board (CAB) to help develop and guide the study to make the priority-setting process better informed and offer relevant choices [47]. Community groups often helped review the recruitment materials and methods, assisted with recruitment and helped overcome recruitment problems, led meetings, and disseminated information to community stakeholders. Other information sources used to design the priority-setting exercises included literature reviews, informal and formal discussions, surveys, and adaptations or follow-ups of other studies.

#### Theme 5: Planning for Implementation

Plans for translating the identified priorities to actual research through policies and funding were hinted at in 21 studies (45%). However, a limited number mentioned who would implement these priorities and how (*n* = 15, 32%) nor reported plans to convert research priorities into implementation projects or strategies. Some examples included the research exercise used to gather targeted recommendations to culturally enhance and adapt autism mental health intervention for Latino families for use by therapists, who were also represented in the priority-setting process [48]. In another study, a consensus project using Aboriginal culture, values, and approaches was developed to gather research priorities for expanding e-health [45]. Additionally, the research questions prioritised using a world café approach were used to guide the development of future research proposals in participatory methods for research prioritisation in primary care [41].

#### Theme 6: Criteria

Criteria used to focus the priority-setting exercises to ensure that important considerations were not overlooked, were outlined in 39 studies (83%). Most of the activities focused on identifying the magnitude of a health problem, e.g., using the needs assessments approach [29, 31, 49]. Some other primary criteria included supporting research financing projects [50, 51], a roadmap for community-engaged practice and research, including identifying barriers and facilitators [41–43, 48, 52], and identifying priorities for specific issues such as pregnancy and child health [24, 28]. Mental health studies explored the feasibility of the priority-setting partnership processes reaching and equitably representing a wide range of patient and professional views [48, 53], how individual priorities changed after group deliberation [54], and evidence to guide the development of tools and approaches [30, 39].

#### Theme 7: Methods for Deciding on Priorities

The approach for deciding on priorities was described in all but two studies, where the approach used was not explicitly reported [55, 56]. Studies adopted either a consensus-based approach (*n* = 27, 57%), a metrics-based approach (i.e. pooling individual rankings of options provided) (*n* = 11, 23%), or a combination of both approaches (*n* = 7, 2%). Surveys, focus groups, interviews, Delphi, and nominal group techniques were the most common methods for deciding priorities. Most quantitative studies used a metrics-based survey or Delphi techniques, while several qualitative studies used consensus-based approaches in combination with thematic analysis to identify priority themes.

#### Theme 8: Evaluation

No studies reported any data on the longer-term impact of priority setting exercises or how long-term impacts will be monitored. One national priority setting, however, made suggestions for plans, strategies, or suggestions to evaluate impact, including an assessment of policy brief submissions and the range of priority domains [50]. Some studies also included recommendations on the need to review the priorities in other related future studies [26, 41, 57].

#### Theme 9: Transparency

Clarity on how priorities were set was provided by all except six studies. Most studies explained how the priorities were established and who was involved when describing the participant characteristics and roles, but the details and representation varied across studies [27, 39, 58, 59]. Some studies with mixed participant groups (e.g., community members and health and social care providers) sometimes presented separate priorities suggested by the different groups [25, 42, 51]. Evaluation of the priority-setting process within the studies was limited, but most included a summary of challenges identified during the priority-setting exercise. Challenges were often related to general community engagement issues in research, e.g., literacy, poor involvement and resource constraints [29, 38, 49, 52, 60]; this was sometimes unique to specific types of research areas such as genomics [60], and such studies advised on caution related to the generalisability of the results [31, 48, 52, 61, 62]. Studies also shared the role of the researcher and the potential influence this may have had, including potential participant selection bias [31, 58] and approaches to mitigating the effect of lack of involvement [46].

### Research Priority Setting Good Practices

This review also explored good practices adopted within the included studies when conducting the research priority exercise. These particularly included approaches to increase participation and representation in the priority-setting exercise and transparency in determining priorities, their order, and impact. Overall, the studies agreed that increasing participation was critical to reducing the overall burden of health disparities and decreasing the pressure on healthcare services [55].

The study by Wapau et al. outlined the differences between institution and community-driven research and presented recommendations for a local approach to health research, which included the need to present the findings at community and outside meetings, report up the line, publish the results, and look for ways to implement the answer [63]. Another study presented core attributes that participants considered as facilitators or barriers when engaging youth in research, and this included the importance of using culturally and contextually appropriate research methods, appropriate researchers, and the inclusion of parents in youth health research [64]. The same study also reported that participants highlighted approaches that need to be cognizant of intergenerational dimensions of topics [64], emphasising how building trust was particularly important in research. Related to this, Yan et al. shared that storytelling as a patient engagement approach can build trust in the patient-research partnership, stating that this helped ensure patients were meaningfully engaged throughout the process and it could also capture the diversity of patient experiences and perspectives [62]. Other measures suggested for genuine and meaningful engagement of patient stakeholders included patients being closely involved throughout the research topic generation and prioritisation process and patient stakeholders having the decision-making power. This required providing more general knowledge on research and mechanisms to provide information on researchers and the studies.

Studies also shared suggestions for meaningful engagement with marginalised communities when choosing research project topics, objectives and intervention design for specific health areas, e.g., mental health needs [46, 49]. Collaborative and consensus-based facilitation approaches were emphasised as effective methods for prioritising community-based knowledge and expertise in setting priorities and direction [53]. For instance, examples of good practice included collaborating on new research projects, advocating for increased refugee partnership in refugee research [65], and using a multi-disciplinary steering group with a representative mix of professional stakeholders, faith, parents, voluntary and community sector representatives, and lay representatives [24]. There were also compelling suggestions on the need to share information through community meetings, researchers being visible in the community, educating the community on the “nature” of research, and being responsive to community-preferred modes of research dissemination [44].

Resource availability was a critical factor, and the study by Zeh et al. outlined vital resources for engaging with ethnic minority participants [66]. This included the need for multilingual services and health link workers, cultural competency training for staff, easy access to translators and interpreters, regular updates, varied information sources (e.g., leaflets, posters, audio-tapes), easy access to community diabetes specialists, and having encompassing health services within research study packages (e.g., for diabetes research to include phlebotomy service, chiropody/foot care, better signposting for self-management, home care visits, dietitian, and healthy lifestyle courses). However, with the general issue of resource constraint, one study suggested that deliberative consideration of research funding priorities produces changes in how individuals prioritise research spending across different areas; these changes reflect individual learning from the exercise and group members [67].

For assessing suggested priority topics from diverse participants, Alotaibi et al. presented six criteria to be considered for scoring the selected research topics (appropriateness, relevance, feasibility, impact of research outcome, opportunity to strengthen collaboration with partners, and urgency) [50]. Furthermore, it was stated that outcome indicators needed to be valid and easily interpretable. Some suggested outcome indicators of research priorities included school literacy, school attendance, and life expectancy for research related to children and young people, as well as referrals to secondary care for research on specific health conditions [51].

## Discussion

This review summarises health and social care research priority-setting studies that included ethnic minority population participants. A global appraisal approach was adopted to present a broad perspective of the involvement of ethnic minorities across different regions and toward calling attention to the need for increasing active pre-research involvement in all countries. The 47 included studies were from 12 countries and covered various population groups and disease areas, spanning from 2010 to 2023, with a significant increase in the number of studies in the last 10 years. Still, the number of studies is considerably low from a global perspective, showing an overall lack of evidence and the need to advocate for more ethnic minority inclusion in research priority settings. Health conditions included cancer, diabetes, obesity, and mental health, while social-related health research of interest included the need to explore the social, cultural, and environmental determinants of health. Most of the studies had a more public health focus and  there was little evidence of biomedical research involvement. For the methodologies used, a group consensus-based approach was the main technique used to gather the priorities. Assessment of the nine priority-setting themes showed that the strengths in most studies were the context (except evidence of the political and economic environmental context) and methods used to conduct the exercises. The evaluation of established priorities was the weakest component, which was not addressed in any of the studies. Our findings suggest that information and monitoring of ethnic minority population engagement in health research priority is lacking in many geographical and health topic areas. These findings are similar to those of other review studies, including one on ethnic minority health research priority setting [14]. In contrast to our review, previous reviews included community members, health and social care professionals, and experts such as ethnic minority health service providers, academics, researchers, and healthcare practitioners [14, 15]. Our primary focus on community members aimed to identify the involvement of local community non-experts towards understanding priority setting actions in alignment with community needs.

The studies identified were predominantly from four countries (USA, UK, Australia, and Canada), indicating poor reporting or involvement of ethnic minorities in most countries. Similar country representation has been identified in other reviews [68]. A previous review narrowed the investigation to priority settings in selected high-income countries, but ethnicity details were not reported [69], thus emphasising the need for this current review to present the global view of ethnicity representation. Similarly, the range of health topics in the included studies was also limited, further highlighting the lack of ethnic minority community members in several health research areas. With the continued improvement of reporting ethnicity enrolment, much effort and innovative thinking is still needed to engage ethnic minority groups in pre-research investigation, particularly for biomedical, clinical, and social care research, as this is needed to understand the barriers to enrolment among these communities with low participation rate in clinical trial studies [70, 71]. For instance, mistrust of the medical systems might be addressed with culturally sensitive and open community engagement, bridging the gap in willingness to participate in health-related research [71, 72]. Literature has also suggested that if offered the opportunity to participate in research, ethnic minorities are as willing to participate as white people are [70, 73–75]. However, information needs to be provided in an acceptable context to effectively increase awareness and reduce mistrust in research, which is a common barrier to willingness to participate [72, 73].

Furthermore, although this study focused on research participation based on ethnicity, other factors, such as socioeconomic deprivation, sociopolitical influences, cultural layout, and country economics, need to be considered, and the contextualisation of these factors will differ across different country [76]. Generally, the economic status of a country influences the funding allocation for research, and funders often determine the priorities for health research with minimal public involvement [77, 78], which determines the possibilities of conducting robust priority setting exercises with adequate population group representation. Even in the presence of inclusive research priority setting activities, there are still several other community and individual barriers ethnic minorities face before participation [79]. For instance, some socio-political issues related to willingness to participate include systemic and structural racism and discrimination, little to no policy representation, and migration legal status, and these factors influence individuals’ willingness to engage in research [80]. Ethnic minority groups also often experience disparities in healthcare access and quality and their distrust of healthcare systems is often due to low cultural competencies of health service providers and researchers, and these further act as deterrents to involvement [1]. So, while ethnicity is an important factor overall, comprehensive research participation and engagement strategies are essential, and multiple cultural factors must be considered. Such exploration need to be tailored to different geographical locations and communities for adequate contextualisation for generating more relevant findings for each setting; this way, research is driven by country level priorities [78, 81].

None of the included studies addressed all the good practice principles as proposed by the checklist. This finding showed that the studies did not explore or report all the recommended priority-setting process components. Good research priority practices identified in this review for increasing participation included a community advisory board and local approach to health research, including a multi-disciplinary steering group and designing activities according to different cultural frameworks and preferences [63]. Consideration of the broader views and cultural dimensions helped develop and guide the study to make the priority-setting process better informed. Also, considering cultural and contextual dimensions surrounding the issues ensures more meaningful engagement. The use of collaborative partnership and consensus-based facilitation allowed communal reflection and individual learning from the exercise and members of the group. A few studies used a clear structure or framework to guide the priority setting and implementation process, showing transparency and reproducibility. Overall, a successful approach relies on engaging and influencing. In addition, demographic representation and participant involvement are essential; however, most studies included in this review had more female participants. High-quality priority-setting exercises require appropriate, balanced gender representation, as this is crucial for research and understanding the broader effects of different health conditions.

Interest-holder (i.e. groups with legitimate interests) involvement was an indispensable part of the research prioritisation process and was used in several studies [82]. Working with selected representatives of ethnic minorities in priority settings, including various organisations and other service providers who work with them, increased the wider involvement [83]. Hence, conducting a stakeholder mapping exercise before commencing a health priority-setting exercise is crucial. The role of stakeholder community representatives can include providing a prior opinion to the approach and design, providing evidence or being a part of the group that decides on priorities [84]. Although our review focused only on public contributions from community members who were not health, social care, and research professionals, in addition to that group, including different sectors and providers such as civil society, policymakers, funders/donors, and the private sector can further enrich the priority-setting conversations [21].

Resources that enhanced the experiences included cultural competence training for staff, language translation and easy access to translators and interpreters, varied information sources, and community-tailored data-gathering approaches such as storytelling, building trust, and giving community decision-making power. Also, group-based approaches appeared to be the most acceptable approach. The advantages of group approaches include quicker, more cost-effective, more diverse perspectives from group dynamic interactions and more flexibility in priority exploration. The gaps in evaluating and implementing the priorities identified were a significant weakness. This shortfall might not be limited to only studies with ethnic minority populations. However, in this context, the gap highlights that ethnic differences have not been paid sufficient attention throughout the research life cycle process, and this can give rise to inequalities, as ethnicity-related social and economic factors are important for understanding health needs and inequalities [85]. Given the increasing diversity in most countries, especially in Europe, it is imperative for health and social care research to include ethnic minority groups in pre-research actions such as priority-setting activities, and doing so effectively needs the implementation of effective and acceptable strategies.

### Strengths and Limitations

The strengths of this review include the description of reported health and social care research priorities in ethnic minority populations, a broad search strategy, a wide range of databases, and the application of a comprehensive methodological framework to evaluate processes.

This review adhered to the PRISMA-ScR reporting guidelines and a rigorous pre-specified protocol, thus limiting the potential for bias. Also, global consideration and comprehensive searching of multiple electronic databases broaden the search scope. On the other hand, limitations included the uneven representation of biomedical research and different health sectors beyond health care, such as social care and community services. Also, although the review was not restricted to any geographic region, the representation of only 12 countries makes generalisation difficult due to the vast cultural differences in the different countries (e.g., USA vs. Saudi Arabia). The reviews might also have been influenced by the search and focus on only studies in English; thus, we might have excluded research priority studies in other languages with no English translations available. In addition, although a few studies had a poor quality score during the quality appraisal, we did not limit study inclusion based on the quality of the studies or the details presented in the priority setting process; hence, the risk of bias, relevance, comprehensiveness, and credibility of the included studies may vary widely. Furthermore, we did not explore other sociocultural factors, such as faith, belief, and other cultural norms, which may have influenced the implementation and involvement of ethnic minorities in research and priority setting exercises.

### Implications for Research and Practice

The review aimed to identify and evaluate the current evidence related to the involvement of ethnic minority community members in health and social care priority–setting exercises, as well as identify good practices for improvement. From our findings, we observed a significant need to broaden the range of health and social care research areas involving ethnic minorities in the priority setting, and more countries need to start exploring this. Also, priority-setting studies need to be guided by standard frameworks or clearly adapted guidelines to encourage efficient planning, implementation, and replicability of the process. Selected guidelines should also align with local and national strategic agendas for easy adoption and enhancing policy impact. The consideration of these points will improve the potential for generalisability of the findings.

Research approaches should include consideration of the context and culture of the target population and will be best through a multi-dimensional consideration that includes using various stakeholders to increase awareness, reduce distrust, and enhance reaching broader ethnic minority populations. It is becoming increasingly recommended that patients and public involvement and engagement (PPIE) be shown in all forms of human research; thus, this review might be important beyond public health research and relevant in planning biomedical and other non-healthcare setting research. Furthermore, more attention is required for implementation and reporting evaluations of the priorities after they have been identified and used to identify research decisions. Evidence has also shown that sometimes research designing activities with ethnic minorities makes them recall previous experiences, and researchers often do not always report the findings on the impacts of their contribution, which influences their willingness to participate [15]. Seeing the policies, completed research studies and observable changes in the community that were spawned by these exercises can be empowering to the community, even if it takes a long time.

Initial findings from this review was used in implementing the priority-setting component of this NIHR Represent Study [16, 86]. This produced a positive and impactful experience when engaging with ethnic minority community groups. Hence, this review can help inform health authorities, research funders, and policymakers about which health and social care research topics, outcomes, and motivations are of most interest to the ethnic minority population in their regions and countries.

## Conclusion

Although reporting of ethnic minority populations involvement in research has increased over time, their representation in setting the priorities for research is significantly limited. Very few countries reported the involvement of ethnic minority community members in research priority-setting exercises, and the range of health and social care areas where they were involved was also limited. In addition, the evaluation of research priorities, once they were identified, was not followed up on. These gaps can contribute to wider disparities in research direction, participation, observation, interpretation, translation, implementation, and overall impact. To address these challenges, researchers, funders, and policymakers need to proactively consider the good practices identified in this review and strategically implement priority-setting exercises that will improve the willingness and involvement of ethnic minorities in research priority setting and subsequently enhance the quality diversity and representation in future research.

## Supplementary Information

Below is the link to the electronic supplementary material.

ESM 1 (DOCX 116 KB)

## Data Availability

Not applicable for a scoping review.
