# Supplementary material for: Global Insights on the Involvement of Ethnic Minority Populations in Health and Social Care Research Priority Setting: A Systematic Scoping Review
Source: J Racial Ethn Health Disparities. 2025 Mar 13;13(3):1818–29. doi: 10.1007/s40615-025-02377-x (PMC13157404; doi:10.1007/s40615-025-02377-x)
Supplement: Supplementary file 1 — (DOCX 116 KB) [file 40615_2025_2377_MOESM1_ESM.docx]

# Title:

Involvement of ethnic minority populations in health and social care research priority setting: a systematic scoping review

# Supplementary

## Supplementary 1: OVID Medline ethnic minority health and social care research scoping review search

1 exp Ethnic Groups/

2 exp Continental Population Groups/

3 exp Minority Health/

4 exp Minority Groups/

5 (Ethnic and Racial Minorities).mp.

6 (ethnic* or minorit* or race or racial or "racial* minorit*" or "Race Factor*" or "mixed race" or "mixed racial" or BME or BAME or "people of color" or "people of colour" or Black or African* or afro or caribbean* or afrocaribbean* or "afrocaribbean" or Asian* or "black british" or bangladeshi* or indian* or chinese or pakistani* or "indian subcontinent" or "south asian*" or gyps* or "irish traveller*" or roma or arab* or jew* or latin* or hispanic* or european* or refugee* or migrant* or immigrant* or "asylum seeker*" or "indigenous" or "Aborigines").af.

7 1 or 2 or 3 or 4 or 5 or 6

8 ("research priority setting*" or "research prioritisation*" or "research prioritisation*" or "research priorities" or "research agenda*" or "research planning" or "research strateg*" or "research intervention*" or "research development" or "research participation*or research engagement*" or "research involvement*" or "research act*").af.

9 7 and 8 9381

10 limit 9 to (english language and humans and yr="2010 - Current")

## Supplementary 2: Checklist for Health Research Priority Setting

| **Themes** | **Questions** |
| --- | --- |
| 1. Context | 1. Resources |
|  | 2. Focus |
|  | 3. Values or principles |
|  | 4. Health environment |
|  | 5. Research environment |
|  | 6. Political environment |
|  | 7. Economic/ financial environment |
| 2. Use of comprehensive approach | 8. Process detail |
| 3. Inclusiveness | 9. Participants described |
|  | 10. Representation of expertise |
|  | 11. Representation of sex/gender |
|  | 12. Representation of regional participation |
|  | 13. Relevant health sectors and other constituencies |
| 4. Information gathering | 14. Information and sources used referenced |
| 5. Planning for implementation | 15. Plans for translation of priorities |
|  | 16. Who will implement priorities and how |
| 6. Criteria | 17. Criteria to focus discussion |
| 7. Methods for deciding on priorities | 18. Approach for deciding on priorities |
| 8. Evaluation | 19. When and how to evaluate established priorities |
| 9. Transparency | 20. Clarity on how priorities were set |

***Sources:*** *Viergever 2010, Iqbal, 2021*

## Supplementary 3: Table of included studies characteristics

| **Authors** | **Country(s) of study** | **Health Topic and Scope** | **Study populations** | **Methodology/**  **Framework** | **Main outcome (research Priorities)** | **Quality score** |
| --- | --- | --- | --- | --- | --- | --- |
| Alotaibi et al. 2022^1^ | Saudi Arabia | General healthcare | Non-Saudi (n=712)  Saudi nationals (n=1,540)  Community involvement represented by 26 organizations | E-Delphi  Survey  ***Framework:*** *WHO Health System* | **Top five research priority themes for MoH headquarters’ leaders and health categories** **Ministry of Health Headquarters’ Leaders**: Health system; Public health; Health informatics and E-health; Health education **Physicians:** Critical care medicine, emergency medical services & emergency; Internal medicine & COVID19; Paediatric medicine, paediatric general surgery and paediatric neurology; Obstetrics & gynaecology; General surgery **Dentists:** General dentistry and all related branches; Dental hygiene; Oral and maxillofacial surgery; Health system, healthcare and hospital administration; Paediatric general dentistry **Pharmacists:** Pharmacy; Health education; Clinical pharmacy; Health system, healthcare and hospital administration; Public health **Health Specialists:** Biomedical technology, clinical biochemistry, clinical laboratory sciences, medical microbiology; Health system, healthcare and hospital administration; Radiology; E-health **Nurses:** Health system; Health education; Critical care medicine; Midwifery and nursing; Community medicine and public health | 18/20 (90%) |
| Asnaani et al. 2022^2^ | USA | Mental health | African American/Black, Latino/a, Native American, Pacific Islander, African immigrants  Phase 1 (n=49)  Phase 2 (n=120)  - Community members  - Providers | Focus group  ***Framework:*** *Community-Informed Framework for Culturally Responsiveness* | **Major Themes Generated** **Community Members**  • Experiences and coping with emotional health  • Stigma or shame regarding emotional health  • Openness to getting treatment  • Barriers to treatment • Impact of politics and racial tension on emotional health  **Community Providers**  • Emotional health issues providers come across • Type of treatment or services offered • Possible modifications to treatment • Stigma or shame present in seeking help • Interest in training | 10/20 (50%) |
| Borthwick et al. 2023^3^ | UK  Australia African Countries | Biomedical research | Community engagement experts (n=4)  Researchers who had engaged communities in their work (n=27) --- African researchers (n=10) | Interviews  *No framework* | **Reasons for community engagement in biomedical research priority-setting** • Epistemic value • Community ownership • Equity • Responsiveness  **Foundations for meaningful engagement** **Environmental:**  • Time • Organisational support and governance frameworks • Funding requirements for community engagement  **Relational** • Embeddedness/existing connections to the community • Diversity amongst the research team • Obtaining gatekeeper support • Trust • Fair processes  **Collective:**  • Self-mobilising  **Individual-researchers** • Understanding of community context • Experience with/literacy in community engagement • Communication skills • Attitudes (respect for communities, recognition of value of community expertise, open-mindedness to different points of view)  **Individual-community** • Able to take a broad perspective • Research literacy, including genomics literacy • Known status within the community • Communication skills and compassion | 12/20 (60%) |
| Bowers et al. 2017^4^ | USA | Community engagement | Hard-to Reach community (n=100) | Meeting attendance  *No framework* | **Factors that turn people away from participating in research:**  1. Passive Language.  2. Specialised use of everyday language.  3. Perceived Tone of Documents.  4.Request for demographic information. | 15/20 (75%) |
| Bowring et al. 2022^5^ | Germany Luxemburg  UK | Parkinson’s | People with Parkinson’s (PwP), research participants, relatives and healthcare professionals (HCP)  Black, Asian and minority ethnic groups (BAME) (11 of 879 responses) | Survey  *No framework* | **Top 10 research priorities for Parkinson’s** 1. What is the best type and dose of exercise (physiotherapy) for improving muscle strength, flexibility, fitness, balance and function in people with Parkinson’s?’ 2. ‘What drug treatments are best for the different stages of Parkinson’s?’ 3. What treatments are helpful for reducing balance problems and falls in people with Parkinson’s? 4. What helps improve the dexterity (fine motor skills or coordination of small muscle movements) of people with Parkinson’s so they can do up buttons, use computers, phones, remote controls etc? 5. What treatments would ensure the medications were equally effective each day (prevented/managed wearing off, variability, on/off states) in people with Parkinson’s? 6. What best treats mild cognitive problems such as memory loss, lack of concentration, indecision and slowed thinking in people with Parkinson’s? 7. What treatments are helpful in reducing tremor in people with Parkinson’s?  8. What is the best treatment for stiffness (rigidity) in people with Parkinson’s? 9. Is it possible to identify different types of Parkinson’s, for example, tremor dominant? And can we tailor treatments best according to these different types? 10. What is helpful for improving the quality of sleep in people with Parkinson’s? | 16/20 (80%) |
| Bryan et al. 2020^6^ | Canada | Knee replacement surgery | South Asian patients and caregivers Healthcare professionals  Patients (n = 31)  Caregivers (n = 5)  Clinicians (n = 27) | Focus groups Modified Delphi survey  ***Framework:*** *Canada’s Strategy for Patient-Oriented Research (SPOR)* | **25 priority research topics identified with 15 having "strong support"**  **Top priorities both for patients and caregivers and for clinicians were**  (1) promoting exercise following surgery and (2) self-management after hospital discharge. One of the highest ranked topics for patients and caregivers was improving knee implants. Patients and caregivers prioritized research on promotion of exercise and self-management following surgery and improvement in knee implants.  **15 research topics with "strong support"**  1. Information on lifestyle changes (e.g., weight loss, diet, exercise) to prevent or delay osteoarthritis 2. Improving other surgical techniques (e.g., arthroscopy) to avoid or delay need for knee replacement surgery 3. Understanding differences between patient and surgeon views on the right time for knee replacement surgery 4. Improving wait times for surgery 5. Effectively managing knee pain before surgery (e.g., medication, complementary and alternative medicine such as massage, homeopathy, diet) 6. Information on lifestyle changes (e.g., weight loss, diet, exercise) before surgery to improve recovery after surgery 7. Improving patient understanding of what to expect during and after surgery 8. Improving knee implants that allow for kneeling, squatting and walking downhill 9. Improving practices for physiotherapy after surgery 10. Exploring the best role of the surgeon after hospital discharge 11. Effective use of pain medication after hospital discharge 12. Effective support from the health care system after surgery 13. Effective self-management after hospital discharge 14. Promoting exercise following surgery 15. Providing effective support to patients with ongoing recovery problems | 13/20 (65%) |
| Bryant et al. 2022^7^ | USA | General health | Hispanic faith community (18 years and over) | Survey  ***Framework:*** *CBPR* | **Ranking of the 5 main community concerns:**  1. Hunger and nutrition 2. Healthcare access 3. Health concerns 4. Access to community resources 5. Community concern | 14/20 (70%) |
| Cartwright et al. 2023^8^ | UK | Children and young people | 12-member multi-disciplinary, multi-ethnic community steering group (involving communities, professional stakeholders) | Workshop  (REPRISE guidelines for priority setting research)  ***Framework:*** *JLA* | **27 research questions of importance to communities for happy and healthy children (in no particular order)** 1. How can we ensure health services are appropriate for community needs and accessed by those that need them? 2. What types of services (voluntary/cultural/youth) are needed to promote health and wellbeing? 3. How can we optimise a healthy diet? 4. What are the barriers to a healthy lifestyle (individual, community, structural)? 5. How does the quality of people’s housing affect their health? 6. How does children’s educational experience impact on their health and wellbeing? 7. How can we reduce exposure to pollution? 8. How best can we improve sustainable travel and encourage active travel? 9. How do we encourage children to be physically active? 10. What are the barriers that stop children from being physical active? 11. What elements of a child’s home environment are most important for health and wellbeing? 12. How do family relationships impact on children’s health and wellbeing? 13. How can we ensure their voices are heard and can influence their future? 14. What are the key issues facing children in terms of their mental health and what can we do about them? 15. How does perception of pressure to succeed impact on health and wellbeing? 16. What is childhood and how does it affect health and wellbeing? 17. How can we ensure access/encourage to high quality natural environments? 18. What is needed to understand how to support or improve parenting skills? 19. What is the impact of vaccinations on children’s health (up-to date, barriers, positive messages)? 20. What is important for health and health conditions? 21. What are the barriers (individual, community, organisational environments) that stop people leading healthy lifestyles? 22. What is important for a healthy mouth for children? 23. How do children’s peer and social relationships affect their health and wellbeing? 24. What is the impact of screen time on children’s health? 25. How we can encourage different ages and communities to work together? 26. How does consumerism effect our health and wellbeing? 27. How do we build inclusive environments for children regardless of culture, ethnicity, disability and background? | 11/20 (55%) |
| Chlebowski et al. 2020^9^ | USA | Autism mental health | Latinx parents (n=29) Therapists (n=17) | Interviews with parents  Focus group with therapists  ***Framework:*** *Framework for Reporting Adaptations and Modifications-Enhanced (FRAME)* | **Recommendation to improve intervention effectiveness (using cultural enhancements)**  **Domain 1: Adapting Training for Therapists** • Improving fit for client language • Increasing fit for client culture  **Domain 2: Content and Contextual Modifications** • Improving fit for client language • Increasing client engagement • Improving intervention effectiveness  **Extending the pacing:** Parents recommended that the treatment be targeted and focused on small specific goals. | 13/20 (65%) |
| Dirks et al. 2019^10^ | USA | Cancer | Alaska Native community (n=28) | Focus groups with 28 adult Alaska Native rural community members  *No framework* | **Participants shared interest in engaging in genetic cancer research and suggested ways to improve community engagement in research.** **General Experiences with Research**  - Weighing the experience of being “over-studied” and the importance of community benefit - Lack of follow-up and the importance of continuous community engagement  **Views on Biospecimen Collection** - Access to biospecimen collection results - Getting biospecimen collection results back  **Views on Collection of Family Health History** - Sharing family health history - Medical records - Contacting family members to collect family health history | 12/20 (60%) |
| Erves et al. 2017^11^ | USA | General health | Underrepresented research populations (including African American and Spanish speaking individuals with hearing impairments) (n=117) | Group discussion (Community Listening Sessions - CLS)  No framework | **4 themes illustrating research priorities:** 1. Uncertainties of underrepresented populations regarding research participation  2. Ineffective communication about research opportunities and research findings 3. High priorities for underrepresented populations in research and healthcare 4. Research teams need training in cultural competence and humility.  **Condition areas of priority:** cancer, Alzheimer’s disease, cardiovascular disease, and diabetes. | 13/20 (65%) |
| Finer et al 2018^12^ | UK | Diabetes | 2587 respondents  - whom 18% were from black, Asian and minority ethnic groups. | Survey  ***Framework:*** *JLA* | **What questions about Type 2 diabetes would you like to see answered by research?**  --- Priority ranking for all groups (PWDC, HCPs, BAME), *(# Ranking assigned by BAME)*  1. Can Type 2 diabetes be cured or reversed, what is the best way to achieve this and is there a point beyond which the condition can’t be reversed? **(#1)** 2. How do we identify people at high risk of Type 2 diabetes and help to prevent the condition from developing? (#50) 3. What is the best way to encourage people with Type 2 diabetes, whoever they are and wherever they live, to self-manage their condition, and how should it be delivered? **(#40)** 4. How do stress and anxiety influence the management of Type 2 diabetes and does a positive mental wellbeing have an effect? **(#6)** 5. How can people with Type 2 diabetes be supported to make lifestyle changes to help them manage their condition, how effective are they and what stops them from working? **(#14)** 6. Why does Type 2 diabetes get progressively worse over time, what is the most effective way to slow or prevent progression and how can this be best measured? **(#10)** 7. Should diet and exercise be used as an alternative to medications for managing Type 2 diabetes, or alongside them? **(#6)** 8. What causes nerve damage in people with Type 2 diabetes, who does it affect most, how can we increase awareness of it and how can it be best prevented and treated? **(#3)** 9. How can psychological or social support be best used to help people with, or at risk of Type 2 diabetes, and how should this be delivered to account for individual needs? **(#25)** 10. What role do fats, carbohydrates and proteins play in managing Type 2 diabetes, and are there risks and benefits to using particular approaches? **(#13)** | 14/20 (70%) |
| Franck et al. 2018^13^ | USA | Maternal health  (Pre-term birth) | BAME women at high risk of preterm birth  (n = 12) | Focus groups  ***Framework:*** *Research Prioritization by Affected Communities (RPAC) framework* | **Top 9 Priority Research topic** 1. Stress during pregnancy and its impact on women and infants 2. Standards for monitoring and care of women with high-risk pregnancies 3. Impact of employment and insurance coverage on care and outcomes 4. Safety of drugs and substances during pregnancy and for newborns 5. Provider-patient communications and decision-making 6. Breastfeeding support 7. Standards for referral of women and families to social services 8. Causes and prevention of Sudden Infant Death Syndrome (SIDS) 9. Support for mothers and babies at home | 10/20 (50%) |
| Goold et al. 2017^14^ | Australia | General health (Patient-Centered Outcomes Research) | Ethnic minority and medically underserved communities (18 to 83 years) Academic and community partners  (n = 183) | Focus groups Interviews Survey  *No framework* | **Individuals and groups prioritized research on:** 1. Quality of life,  2. Patient-doctor relations 3. Access 4. Special needs  5. Compare approaches  - Black participants were less likely to prioritize research on causes of disease, new approaches, and compare approaches than White participants | 14/20 (70%) |
| Goold et al. 2018^15^ | USA | General health | Ethnic minority and medically underserved communities (47 groups, n = 519) | Document Review Focus groups Key informant interviews Surveys  *No framework* | **Highest priority topics**: child health research and mental health research.  **Other prioritised topics:** Aging, access, promote health, healthy environment, and what causes disease   - Black/African American or non-white participants more likely to prioritize communication research - Black/African American participants less likely to prioritise mental health research  - Native American and Arab American participants prioritised research on culture and beliefs - Hispanics less likely to prioritize aging research. | 17/20 (85%) |
| Green et al. 2018^16^ | Australia | Cancer | Indigenous people affected by cancer (n = 17)  Health professionals (n = 28)  Individuals in both groups (n = 7) | Focus groups Interviews  ***Framework:*** *National Aboriginal and Torres Strait Islander Cancer Framework* | **Key issues impacting Indigenous patients’ experience of cancer care** • Feeling safe in the system • Importance of Indigenous care providers • Barriers to care • The role of family and friends • Effective communication and education • Coordination of care/navigation of the system and transition between services  **Participants’ views on ‘how to measure’ experiences of care:** face-to-face interview with a trusted person considered as best approach | 13/20 (65%) |
| Iqbal et al. 2022^17^ | UK | Obesity | British Pakistani Women (n=23) | Focus groups  *No framework* | **Women's obesity related concerns:**  **Theme 1: Distrust** − Information given around medication. − Conflicting dietary information given by health professionals. − Women‐only organized physical activities.  **Theme 2: Cultural barriers** − Gender role of the woman. − Lack of culturally appropriate dietary advice. − Lack of understanding of healthy/unhealthy diet. − Lack of culturally appropriate exercise facilities around physical activity. − Conforming to family and community expectations. − Lack of understanding of healthy weight.  **Theme 3: Language barriers** − Lack of understanding. − Inability to read Urdu. − Reluctance to attend weight management services. − Reliance on others for information. | 10/20 (50%) |
| Kreuter et al. 2012^18^ | USA | General health | African Americans (n=>200)  - Organizations offering services (n=25) | Listening session Photovoice Medical records review  ***Framework:*** *CBPR* | **Medical records top 10 diagnoses ranking by gender:** **Males:**  Hypertension, Acute upper respiratory infection, Diabetes, Eye disorders, Teeth disorder, Asthma, Skin rash edema, Dermatophytosis, Atopic dermatitis, Conjunctiva **Females:** Hypertension, Diabetes, Inflammation of cervix or vagina, Eye disorders, Menstrual problems, Teeth disorders, Dermatophytosis, Skin rash edema, Atopic dermatitis, Asthma  **Community-based process, 2 priorities emerged:** 1**. Top priorities of depression and mental health** by focusing on improving access to mental health services (different from epidemiologic data/medical records) 2. N**egative impacts of vacant housing in the neighbourhood**, as the means to promote advocacy and community empowerment to improve environmental and housing conditions (factors known to influence depression and hopelessness) in NPU-V.  **Priority themes from photographs**: trash, construction debris, poor neighbourhood living conditions, vacant housing and loss of community. | 14/20 (70%) |
| Maar et al. 2010^19^ | Canada | Health access (E-health) | Aboriginal council members and community members Regional and provincial, and federal leaders Policy makers  (n=40) | Literature review Indigenous research methods  ***Framework:*** *PAR* | **6 distinct priority topics**  1. Ethical principles for Aboriginal e-health Research 2. Internet-based national information for Aboriginal ehealth initiatives 3. Research related to e-health education and professional development 4. Sustainability 5. Best practices 6. Broader applications and impact of ehealth on Aboriginal culture and communities | 13/20 (65%) |
| MacFarlane et al. 2017^20^ | Ireland USA | Primary care | Community and health care stakeholders (n=63 Ireland and n=55 USA). | Two participatory World Cafés  ***Framework:*** *Adapted conceptual framework for patient engagement* | **Summary of priority issues for further research Ireland** • Improve people’s knowledge and understanding of primary health care services • Develop health promotion activities for chronic conditions  • Focus on the specific needs of certain social groups, e.g. migrants, people with disabilities, older people, men’s health, travellers • Focus on sustainable community, physical space and resources and leadership across the life course • Find out what works well and implement it in practice  • Put the community at the heart of health service planning and delivery  **USA** • Understand people’s perspective regarding primary-care diabetes-related services, and barriers to receipt of care • Focus on the needs of certain social groups (participating immigrant and refugee communities) • Find out what works well and how it could be implemented in practice  • Put the community at the heart of service delivery and planning • Develop a discrete plan to improve local health services, i.e. choose among a set of interventions that improve quality and outcomes of care that can be compared in a future research project • Focus on developing research capacity among participants | 11/20 (55%) |
| Manikam et al. 2017^21^ | UK | Child health | South Asian children and families (16-74 years) Heath care practitioners from various backgrounds (n=62) | Systematic literature review Scoping survey Focus groups  *No framework* | **Community (South Asians, SAs) topics and outcome indicator priorities:** 1. Concordance and shared decision making 2. Primary care access 3. Mental health 4. Obesity and diet 5. Blood and Organ donation 6. Alternative medicine effectiveness 7. Routine health monitoring **Health care practitioners (HCPs) priorities:** -- **Public awareness** on Obesity and diet; Mental health; Healthcare access; ; Vitamin D and rickets; Routine health checks: Allergy and asthma; Dental health; Link between genetic disorders and consanguinity; Domestic violence and safeguarding -- **Research** on Nutrition; Diabetes; Health-care access and health-seeking behaviour; Health education; Parenting methods; Asthma; Dental health; Infectious diseases  - HSPs and SAs prioritized increased research or public awareness on mental health illness, blood and organ donation, obesity, and diet. - Effectiveness of alternative Medicines (not identified by HCPs.) - HCPs identified diabetes, vitamin D and rickets together with parenting methods were important priorities requiring increased public awareness, (but views not shared by SAs. | 17/20 (85%) |
| McMorrow & Saksena 2017^22^ | USA | Refugee Healthcare | Congolese refugee women (n=16) | Semi-structured Interview Photovoice  ***Framework:*** *CBPR* | **Three major themes**:  - Health care system issues - Social support - Daily experiences of health | 13/20 (65%) |
| Mohamed et al 2022^23^ | USA | General | Somali Adults (n=646) | Survey  ***Framework:*** *CBPR* | **Important health issues**  ***For individuals and their families:***  - Health behaviours (including Diet. Exercise) (22.7%) - Diabetes (18.2%) - Hypertension (14.4%) - Weight/obesity (12.2%) - Infectious diseases (e.g. viral hepatitis, influenza, tuberculosis) (4.4%) - Hepatitis B or C (1.9%) - Cancer (3.1%) - Gastrointestinal diseases (e.g. reflux, liver disease) (3.0%) - Heart disease (2.7%) - Musculoskeletal (e.g. arthritis, back, knee pain) (2.5%) - Neurological diseases (e.g. stroke, dementia) (2.4%) - Pulmonary diseases (e.g. asthma) (2.2%) - Mental health (e.g. stress, depression) (2.0%) - Health care (e.g. access to care, cost of care) (1.0%) - Other (e.g. environment, eye disorders) (9.2%)  ***For community*** - Diabetes (22.5%) - Hypertension (18.8%) - Weight/obesity (15.9%) - Health behaviours (including Diet. Exercise) (15.3%) - Other health behaviours (e.g. sleep, smoking) (2.4%) - Infectious diseases (7.9%) - Hepatitis B or C (6.6%) - Mental health (e.g. stress, depression) (4.4%) - Cancer (3.9%) - Heart disease (1.7%) - Substance abuse (e.g. drugs, alcohol) (1.2%) - Other (e.g. environment, asthma) (8.5%) | 11/20 (55%) |
| Montesanti et al. 2022^24^ | Canada | Mental health | Indigenous people (n=53) | Workshop  ***Framework:*** *NGT*  *- Indigenous framework of Two-Eyed Seeing* | **4 themes emerged for setting priorities and directions** a) Understand the realities of Indigenous mental health experience for Indigenous peoples b) Design a holistic and culturally rooted mental health system c) Foster cross-sectoral engagement and collaboration d) Focus on children and youth. | 12/20 (60%) |
| Morris et al. 2017^25^ | Australia | Cancer control | Indigenous cancer survivors and their families, community;  Researchers, health practitioners, advocacy groups  (n=225) | Online survey  ***Framework:*** *National Aboriginal and Torres Strait Islander Cancer Framework* | **Identified research prioritiy themes:**  1. Cancer prevention and early detection  2. Health literacy  3. Culturally appropriate care for Indigenous patients, survivors, and families 4. Cross-cutting themes (partnerships and collaborate with key stakeholders; community engagement and Indigenous ownership) | 15/20 (75%) |
| Park et al. 2022^26^ | USA | Overactive bladder | Total (n=522)  Hispanic/Latino (n=30) Non-Hispanic/Latino (n = 202) Alaska Native (n = 5)  Asian (n = 26)  Native Hawaiian/Pacific Islander (n = 2)  Black/African American (n=16)  White (n = 174)  Other (n = 9) | Survey  *No framework* | **Research prioritization topics** 1. What are the best ways to teach patients with overactive bladder about it? 2. How can doctors use new research to take care of overactive bladder patients? 3. What are the causes of overactive bladder and how can we test for it? 4. What’s the best treatment for me based on my age, race, gender and other medical problems? 5. How can I get my overactive bladder treated fastest? 6. How can things like exercise and meditation help improve overactive bladder symptoms? 7. What are the cheapest treatments for overactive bladder? 8. How can therapy, counselling and support groups be used to help people experiencing overactive bladder? 9. How do patient and doctor expectations determine how well overactive bladder treatment works? 10. What can be done to help patients with overactive bladder be more productive at work?  **Examples of How Research Findings Could Help Improve Patient Care (these are responses to each of the responding numbered questions above) Talking about overactive bladder** 1. Figure out the best way you learn, ie educational materials like brochures, websites, videos or in-person meetings 2. Help doctors stay up to date with new overactive bladder research and treat you in the best way possible. 3. Find the reason you have overactive bladder and better testing to find overactive bladder.  **Overactive bladder care**  4. Doctors might learn that women and men need different types of treatment. 5. Make it easier to get screened for overactive bladder so you can begin treatment or get referred to a specialist. 6. Understand better what changes in my daily habits can help improve my OAB. 7. For example, comparing the cost of surgery (time off work and cost of surgery) to taking a lifetime of medications. 8. Help doctors understand how to use mental health programs or online forums to help patients with overactive bladder. 9. To help make sure treatments meet patient expectations for cure of overactive bladder.  **Work and Disability**  10. Help workplaces improve access to bathrooms and ensuring enough time to go to the bathroom. Think about laws to help employees with overactive bladder. | 10/20 (50%) |
| Payton et al. 2022^27^ | Canada USA | Refugee Healthcare | Research, Evaluation, and Ethics Committee Under the Society of Refugee Healthcare Providers (n=22)  - Includes resettled refugee health care professionals with prior refugee health research experience (n=5) | Survey  ***Framework:*** *Logic Model Framework* | **Top 3 priorities reported by committee for the upcoming year:** (1) Collaborating on new research projects,  (2) Advocating for increased refugee partnership in refugee research, and  (3) Sharing resources about refugee health research and ethics through the listserv.  **The main short-term outcomes include**  (1) Research: Establish priorities for refugee health research, enhance research collaborations among SRHP and committee members, and commitment to equal opportunity for research leadership and partnerships, particularly for refugees and host communities;  (2) Evaluation: Support evaluation of existing refugee health structures and programs via program evaluation, research, and quality improvement initiatives; and  (3) Ethics: Establish ethical framework in refugee health research.   **The main long-term outcomes include**  (1) Research: Foster translation of research findings to clinical, policy, and advocacy work and improve understanding of best and promising practices to achieving health equity among resettled refugee populations;  (2) Evaluation: Guide public health practices among refugee populations; and  (3) Ethics: Guide health equity recommendations related to refugee populations. | 15/20 (70%) |
| Pearson et al. 2019^28^ | USA | Mental Health & HIV (PTSD, Substance Misuse, and HIV) | American Indian tribal women (n=15) | Workshop  ***Framework:*** *Culturally tailored framework - Intervention Mapping (IM) - CBPR* | **Strong agreement from participants on the intersecting risks of trauma, sexual risk behavior, and substance use  Socioecological levels:** Pain and anger around trauma and micro aggressions; and concerns of “broken families” and “intergenerational childhood sexual and physical trauma co-occurring with substance use.”   **Individual level:** PTSD symptoms and post-trauma reaction; behaviors of avoidance and hypervigilance, and reexperiencing trauma memories; Substance misuse and sexual risk behaviors; women expressed little control or autonomy over sex, and a lack of opportunities for personal growth  L**ack of opportunities** identified by community leaders described the burden their women carry and the severe **lack of supportive resources**. | 4/20 (20%) |
| Perrin et al. 2021^29^ | Australia | Diabetes-related foot health disease | Health professionals (n=121)  Consumers (n=72, including carers (n=2), researchers (n=9), industry representatives(n=8)  - Only one participant identified as an Aboriginal or Torres Strait Islander person. | Survey  ***Framework:*** *Modified Delphi aligned with previous JLA approach* | **Final ‘top-10’ research questions** 1. What are the health and cost benefits of providing government-funded multidisciplinary high-risk foot services for optimal management of people with diabetes-related foot disease compared with usual care?  2. What are the most effective treatment options for pain associated with diabetes-related peripheral neuropathy? 3. Whare are the most effective educational programs to improve self-care practice to prevent foot diease in people with diabetes?  4. What are the long-term outcomes (wound recurrence, osteomyelitis recurrence) of osteomyelitis in people with diabetes, when treated conservatively/medically rather than surgically? 5.Can established pheripheral neuropathy be reversed, and if so how?  6.Is there a cost benefit of implementing a program to prevent diabetes-related foot ulcers in high risk populations compared with cost of treating diabetes-related foot ulcers?  7. What smart technology can be integrated into the care of people with diabetes-related foot ulcers that will help improve healing time? 8. Is exercise beneficial in reducing the risk of diabetes-related foot complication? 9. What is the effectiveness of diet supplementation with micronutrients such as vitamins, protein, minerals and amino acids in improving healing rates in people with diabetes-related foot disease or ulcers? 10. Is exercise safe for people with diabetes-related foot complications? | 19/20 (90%) |
| Pratt et al. 2022^30^ | India | Global health  (structural barriers to sharing power with communities) | Indigenous community | Workshop  ***Framework:*** *PAR* | **5 Areas for sharing power (pitfalls and challenges):** • **Scope:** Dual agenda **• Level of participation**: Using PAR as an priority-setting process **• Compensation:** Promoting community organisation control and transparency  **• Space:** Making an invited space into a created space **• Accountability:** Feeding back, Acting on data, Promise keeping. | 12/20 (55%) |
| Purkey et al. 2019^31^ | Myanmar | General health | Organisations representing ethnic minority groups (n=16) | Workshop  ***Framework:*** *NGT* | **Final research priorities identified, in order of ranking** 1. Water quality and contamination (e.g. mining, etc.) 2. Mental health 3. Illegal drug use 4. Under-five malnutrition 5. Pharmaceuticals 6. Antenatal care 7. Food security 8. Non-communicable diseases/hypertension 9. Immunisations 10. WASH 11. Health education 12. Family planning 13. Alcohol consumption 14. Safe delivery 15. Malnutrition among vulnerable populations | 17/20 (85%) |
| Ramirez et al. 2011^32^ | USA | Child health  (Obesity) | Total (n = 313)  Ethnic community members (including Hispanic/Latino, African American and Asian or Pacific Islander)  Academics, researchers, health educators, administrators, managers, clinicians, public health workers, students | Modified web-based Delphi survey  **Framework:**  *Social ecological model approach* | **25 research priorities identified within 5 domains.  Ranking of the 5 main research areas and the top priority for each domains was:** 1. **Family:** engaging Latino families as advocates of childhood obesity prevention initiatives at the community and school levels 2. **Community**: built environment policies involving collaborations with multiple stakeholders to promote physical activity 3. **School** (day care, preschool, grade school, high school): health, nutrition, and active physical education classes as part of the school curriculum 4. **Society**: Policies that subsidize accessibility of healthy foods to improve diet among Latino families 5. **Individual:** programs making physical activity more attractive than watching TV or playing video games | 13/20 (65%) |
| Roura et al. 2015^33^ | Spain | General health (Health status and determinants) | Latin American Migrants (n=84) | Group discussions (Task-oriented visual activities)  ***Framework:*** *Socio-ecological theoretical framework* | **9 Identified themes:** 1. Occupational stress: “working yourself to death” 2. Irregular residence status and discrimination: “sudacas” and “panchitos” 3. An accelerated rhythm of life: “catching stress” 4. Health behaviours: “eat, eat, and eat, because of anxiety” 5. Allergies and skin problems: “from natural to chemical” 6. Sexually transmitted infections: “there is more promiscuity here” 7. Diseases of the poor: “tuberculosis, lice and Chagas” 8. Non-communicable conditions 9. The health system: “ibuprofen and paracetamol”  - Major health concern: Cumulative toll of daily stresses rather than as specific diseases. - Work-related factors, legislative frameworks regulating citizenship entitlements and feeling ethnically discriminated were major sources of psycho-social strain.  - Except for sexually transmitted infections, participants rarely referred to communicable diseases as a concern. | 11/20 (55%) |
| Scorgie et al. 2017^34^ | South Africa | HIV prevention | Internal and cross-border migrants (n=1458) | Surveys Community mapping Client-simulation exercise in local clinics Interviews  ***Framework:*** *Sustainable Livelihoods Approach (SLA)* | **Community-led prioritised needs ranking:** 1. Unemployment  2. Health  3. Poverty 4. Safety 5. HIV 6. Sanitation 7. Housing 8. Water 9. Other (includes: alcohol, shack fires, no toilets or dirty toilets, dirtiness, intermittent or absent electricity, tribalism, frequent death in the community, lack of understanding and information) 10. Education | 14/20 (70%) |
| Shabu et al. 2022^35^ | USA | Cardiovascular Diabetes Cancer Mental health | Black African American (n=427) Hispanic (n=302) White (n=508) | Primary Care Registry enrolment database  *No framework* | **Research interest by ethnic group** **Cardiovascular health:** Black (71.6%), Hispanic (70.8%), White (69.9%) **Diabetes:** Black (46.2%), Hispanic (60.2%), White (45.3%) **Cancer**: Black (31.0%), Hispanic (44.2%), White (42.5%) **Mental health:** Black (54.5%), Hispanic (49.5%), White (63.0%) | 12/20 (60%) |
| Spurling et al. 2017^36^ | Australia | General health | Urban Aboriginal and Torres Strait Islanders (n = 21) | Interviews  *No framework* | **3 themes emerged, to be translated into research priorities:** 1. Complex, interrelated intergenerational nature of health involving social, cultural, and environmental determinants of health 2. Ambivalence about health assessments 3. Community strength | 14/20 (70%) |
| Stone et al. 2020^37^ | USA | Mental health | Total (n= 61)  - Community residents (n=26), 88% minorities  - Service providers (n=23):  - 12 Mental program directors (n=12) | Focus groups Interviews  *No framework* | **Three major themes were identified as systems-related specific barriers to seeking mental health services:**  (1) difficulty navigating the health system (2) language barriers, and  (3) dearth of culturally competent providers.  **Two major themes were identified as psychosocial barriers to seeking care:** (1) mental health stigma  (2) mental health literacy and non-Western notions of mental health. | 13/20 (65%) |
| Tamlyn et al. 2023^38^ | USA | Engagement in research participation | Total (n=38)  Black (30%) Latino (25%) | Interviews  ***Framework:*** *Consolidation Framework for Implementation Research (CFIR)* | **Preferences for engagement in research participation** 1. Wide variation in research recruitment preferences  2. Logistical complexity negatively impacts willingness to participate 3. Risk contributes to hesitation towards participation in research studies.  4. Personal/community benefit, interest in the study topic, and compensation serve as motivators for research participation.  5. Continued participation despite reported shortcomings of informed consent process.  6. Mistrust could be overcome by relationship with or credibility of information sources. | 16/20 (80%) |
| Turin et al. 2021^39^ | Canada | Health Care Access | Bangladeshi-Canadians  (n=432) | Survey  ***Framework:*** *CBPR*  *- Community-engaged integrated knowledge translation (iKT) approaches* | **10 solution-oriented research priority topics (by relative weighted ranking)** 1. Lack of resources  2. Lack of knowledge  3. Health care cost  4. Workplace-related barriers  5. Lack of interest of caregivers/system providers  6. Lack of continuity across providers  7. Travel/transportation barriers  8. Cultural differences/preferences/perception  9. Language barriers 10. Perceived discrimination | 11/20 (55%) |
| Valerio et al. 2016^40^ | USA | Chronic Pain | Hispanic (hard-to-reach communities) - People who experienced chronic pain (35–75 years) and their caregivers (n=55) | Group discussion  ***Framework:***  *NGT (Using mixed methods analysis)* | **Importance of 8 categories of needed pain management services and support:** 1. Professional Chronic Pain Support 2. Nutrition Program 3. Massage Therapy 4. Education/Outreach 5. City Improvements/ Transportation 6. Non-Professional Chronic Pain Support 7. Water Therapy 8. Exercise/Fitness Facility | 11/20 (55%) |
| Varese et al. 2023^41^ | UK | Sexual Violence and Abuse | Survivors of sexual abuse and violation (n = 121) Professionals (n=36) Professionals with lived experience (n=66)  **Ethnicities:** White (n=194), Asian/British (n=3), Black/British (n=3), Mixed race (n=7), Other (n=7), Prefer not to say (n=9) | Survey (online)  ***Framework:*** *JLA* | **Top 10 priorities for sexual violence and abuse research** 1. From the perspective of survivors of sexual violence/abuse, what does recovery involve, what outcomes do they value and what factors can promote these outcomes? 2. How can survivors of sexual violence/abuse who identify as people of colour or as members of black, Asian and minority ethnic groups be best supported? 3. How can access to high-quality psychological therapies for survivors of sexual violence/abuse be improved? 4. What interventions with the general public could reduce misconceptions and stigmas about sexual violence/abuse and their consequences on survivors of sexual violence/abuse? 5. How can the process of police reporting and police investigation best support survivors of sexual violence/abuse and avoid retraumatisation, distress and victim-blaming attitudes? 6. What support is most helpful to and valued by survivors of sexual violence/abuse themselves? 7. How can mental health services and physical healthcare services that are likely to come into contact with survivors of sexual violence/abuse (for example, dental care, general practice, accident and emergency, intimate healthcare and pregnancy termination settings) become more ‘trauma informed’ to best support survivors and prevent retraumatisation? 8. How does involvement in the criminal justice system impact survivors of sexual violence/abuse (for example, their emotional and psychological well-being), and what support do they need during and in the aftermath of criminal justice proceedings? 9. How can support be more accessible, inclusive and effective for survivors of sexual violence/abuse who identify as LGBTQ+? 10. How can survivors of sexual violence/abuse be supported to report sexual violence/abuse that happened many years ago, and what services should be offered to help them recover? | 14/20 (70%) |
| Wapau et al. 2022^42^ | Australia | Community-driven health research | Aboriginal, Torres Strait Islander and non- Indigenous people (n=24) | Workshops  ***Framework:*** *Torres Strait Cultural framework* | **The local approach to health research: What to do with the results** • Feedback to individuals, families, the community, contributors and funders  • Targeted feedback: policy-makers  • Conferences and other meetings  • Internal reports  • Writing a paper: participants will write one or more brief ‘publishable’ reports together  • Explore ways to translate the findings into community health benefits; focus on sustainability  • Setting up a long-term research agenda with sustainable ways to cultivate local research capacity | 12/20 (60%) |
| Watson et al. 2013^43^ | USA | Mental health | Poor and Underserved Latinos - Latino lay health promoters - Key informants (n=30) | Focus groups Key informant interviews Workshop  ***Framework:***  *CBPR* | **Main issues:** 1. Mental health stigma 2. Consequences of immigration- related stress 3. Violence and alcoholism 4. Concerns about psychotropic medications. - In addition common practice of using traditional remedies and medications brought from countries of origin | 15/20 (75%) |
| Wong et al. 2015^44^ | New Zealand | Youth health | Asian youth (18–24 years) (n=15) | Focus groups  *No framework* | **Priorities for research** 1. Cultural differences and identity 2. Racism and discrimination 3. Access mental health issue  **Approaches to research** 1. Culturally and contextually appropriate research methods 2. Appropriate researchers 3. Inclusion of parents in youth health research | 10/20 (50%) |
| Yan et al. 2020^45^ | USA | Breast cancer | African American women breast cancer survivors (20 -45 years) (n=100) | Stakeholder group meetings Storytelling workshops and conference  ***Framework:***  *Innovative patient-centred storytelling approach* | **5 identified priority topics:** 1. Addressing access to care and insurance coverage, such as eligibility for early detection (based on age), access to quality mammography, referrals to genetic counselling 2. Providing support and educational programs for caregivers, friends, and family members of breast cancer survivors 3. Managing breast cancer treatment side effects (tiredness, headaches, pain and numbness, lymphedema, bone loss and osteoporosis, heart problems, menopause, sexual difficulties, infertility, chemo brain), and addressing their impact on quality of life and minimizing the impact of financial hardship experienced by cancer survivors 4. Enhancing patient understanding of treatment options (standard of care, clinical trials) and right to seek second opinions 5. Incorporating spirituality and positive thinking during and after treatment | 14/20 (70%) |
| Zeh et al. 2018^46^ | UK | Diabetes | General practices (n=34): ethnic minority staff (n=108), White British staff (n=408) | Survey  ***Framework:***  Culturally-Competent Assessment Tool (CCAT) | **Local services likely to support staff to deliver better services to minority groups with diabetes (by no. of GP practices who voted). 6 GP practice votes:**  DM information leaflet, posters, audio-tapes  **5 GP practice votes:**  Structured education programmes of ethnic minority groups  **3 GP practice votes**:  • Chiropody/foot care withing practices • Practice dietitian • Easy access to translators and interpreters  **2 GP practice votes** • Multilingual health link workers • Health lifestyle courses  **1 GP practice vote** • Phlebotomy services • Better signposting of diabetes self-management • Culturally-competency training for staff • Home care by practice • General Practitioner with a Special Interest (GPwSI) in diabetes • Community nurses with ethnic minority groups languages • Regular updates in diabetes • Easy access to community diabetes specialists • Multilingual services | 15/20 (75%) |
| Zumeta et al. 2022^47^ | Spain | Engagement in research | Total (n=204)  - Foreigners and immigrants (n=55)  - Born in Spain (n=149) | Survey (online and outdoor)  ***Framework:***  *CBPR* | **Conducted Multiple Mediation Model of Well-Being and Group Efficacy on the following:** **Direct effects** • Quality of participation on self-transcendent emotions  • Quality of participation on shared flow  • Self-transcendent emotions on well-being  • Shared flow on well-being  • Quality of participation on well-being • Age on well-being  • Gender on well-being   **Indirect effects** • Via self-transcendent emotions  • Via shared flow   **Total effects** • Quality of participation on well-being  • Age on well-being • Gender on well-being | 7/20 (35%) |

**Note:** Community based participatory research (CBPR), Consolidation Framework for Implementation Research (CFIR), James Lind Alliance (JLA), Nominal group technique (NGT), Participatory Action Research (PAR), Sustainable Livelihoods Approach (SLA)

## Supplementary 4: Quality Assessment of included studies

| **Authors (name, year)** | **1. Context** | | | | | | | **2. Use of comprehensive approach** | **3. Inclusiveness** | | | | | **4. Information gathering** | **5. Planning for implementation** | | **6. Criteria** | **7. Methods for deciding on priorities** | **8. Evaluation** | **9. Transparency** | **Quality score** |
| --- | --- | --- | --- | --- | --- | --- | --- | --- | --- | --- | --- | --- | --- | --- | --- | --- | --- | --- | --- | --- | --- |
|  | **1. Resources** | **2. Focus** | **3. Values or principles** | **4. Health environment** | **5. Research environment** | **6. Political environment** | **7. Economic/ financial environment** | **8. Process detail** | **9. Participants described** | **10. Representation of expertise** | **11. Representation of sex/gender** | **12. Representation of regional participation** | **13. Relevant health sectors and other constituencies** | **14. Information and sources used referenced** | **15. Plans for translation of priorities** | **16. Who will implement priorities and how** | **17. Criteria to focus discussion** | **18. Approach for deciding on priorities** | **19. When and how to evaluate established priorities** | **20. Clarity on how priorities were set** |  |
| Alotaibi et al. 2022 | Yes | Yes | Yes | Yes | Yes | Yes | Yes | No | Yes | Yes | Yes | Yes | Yes | Yes | Yes | Yes | Yes | Yes | No | Yes | **18/20 (90%)** |
| Asnaani et al. 2022 | Yes | Yes | Yes | No | Yes | No | No | Yes | No | Yes | No | No | No | Yes | No | No | Yes | Yes | No | Yes | **10/20 (50%)** |
| Borthwick et al. 2023 | No | Yes | Yes | No | Yes | No | No | Yes | Yes | Yes | Yes | Yes | No | Yes | No | No | Yes | Yes | No | Yes | **12/20 (60%)** |
| Bowers et al. 2017 | Yes | Yes | Yes | No | Yes | No | No | Yes | Yes | Yes | Yes | No | Yes | Yes | Yes | Yes | Yes | Yes | No | Yes | **15/20 (75%)** |
| Bowring et al. 2022 | Yes | Yes | Yes | Yes | Yes | No | No | Yes | Yes | Yes | Yes | Yes | Yes | Yes | No | Yes | Yes | Yes | No | Yes | **16/20 (80%)** |
| Bryan et al. 2020 | No | Yes | Yes | Yes | Yes | No | No | Yes | Yes | Yes | No | Yes | Yes | Yes | No | No | Yes | Yes | No | Yes | **13/20 (65%)** |
| Bryant et al. 2022 | No | Yes | Yes | Yes | Yes | No | No | Yes | Yes | Yes | Yes | Yes | No | Yes | Yes | Yes | No | Yes | No | Yes | **14/20 (70%)** |
| Cartwright et al. 2023 | Yes | Yes | Yes | No | Yes | No | No | Yes | No | Yes | No | No | Yes | Yes | No | No | Yes | Yes | No | Yes | **11/20 (55%)** |
| Chlebowski et al. 2020 | Yes | Yes | Yes | No | Yes | No | No | Yes | Yes | Yes | Yes | No | Yes | Yes | No | No | Yes | Yes | No | Yes | **13/20 (65%)** |
| Dirks et al. 2019 | Yes | Yes | Yes | Yes | Yes | No | No | Yes | Yes | No | Yes | No | Yes | Yes | No | No | Yes | Yes | No | No | **12/20 (60%)** |
| Erves et al. 2017 | No | Yes | Yes | Yes | Yes | No | No | Yes | Yes | Yes | Yes | Yes | No | Yes | No | No | Yes | Yes | No | Yes | **13/20 (65%)** |
| Finer et al 2018 | Yes | Yes | Yes | No | Yes | No | No | Yes | Yes | Yes | No | No | Yes | Yes | Yes | Yes | Yes | Yes | No | Yes | **14/20 (70%)** |
| Franck et al. 2018 | No | Yes | No | Yes | Yes | No | No | Yes | Yes | Yes | Yes | No | No | Yes | No | No | No | Yes | No | Yes | **10/20 (50%)** |
| Goold et al. 2017 | No | Yes | Yes | Yes | Yes | No | No | Yes | Yes | Yes | Yes | No | No | Yes | Yes | Yes | Yes | Yes | No | Yes | **14/20 (70%)** |
| Goold et al. 2018 | No | Yes | Yes | Yes | Yes | Yes | Yes | Yes | Yes | Yes | Yes | Yes | No | Yes | Yes | Yes | Yes | Yes | No | Yes | **17/20 (85%)** |
| Green et al. 2018 | Yes | Yes | Yes | No | Yes | No | No | Yes | Yes | Yes | No | Yes | Yes | Yes | No | No | Yes | Yes | No | Yes | **13/20 (65%)** |
| Iqbal et al. 2022 | Yes | Yes | Yes | No | Yes | No | No | Yes | Yes | No | No | No | No | Yes | No | No | Yes | Yes | No | Yes | **10/20 (50%)** |
| Kreuter et al. 2012 | No | Yes | Yes | Yes | Yes | No | No | Yes | Yes | Yes | No | Yes | Yes | Yes | Yes | No | Yes | Yes | No | Yes | **14/20 (70%)** |
| Maar et al. 2010 | No | Yes | Yes | No | Yes | No | No | Yes | Yes | Yes | No | Yes | Yes | Yes | Yes | No | Yes | Yes | No | Yes | **13/20 (65%)** |
| MacFarlane et al. 2017 | Yes | Yes | Yes | No | Yes | No | No | Yes | No | Yes | No | No | Yes | Yes | No | No | Yes | Yes | No | Yes | **11/20 (55%)** |
| Manikam et al. 2017 | No | Yes | Yes | Yes | Yes | No | No | Yes | Yes | Yes | Yes | Yes | Yes | Yes | Yes | No | Yes | Yes | No | Yes | **17/20 (85%)** |
| McMorrow & Saksena 2017 | Yes | Yes | Yes | Yes | Yes | Yes | No | Yes | Yes | No | No | No | No | Yes | Yes | No | Yes | Yes | No | Yes | **13/20 (65%)** |
| Mohamed et al. 2022 | Yes | Yes | Yes | No | Yes | No | No | Yes | Yes | No | Yes | No | No | Yes | No | No | Yes | Yes | No | Yes | **11/20 (55%)** |
| Montesanti et al. 2022 | Yes | Yes | Yes | No | No | Yes | No | Yes | No | Yes | No | Yes | Yes | Yes | No | No | Yes | Yes | No | Yes | **12/20 (60%)** |
| Morris et al. 2017 | No | Yes | Yes | No | Yes | No | Yes | Yes | Yes | Yes | Yes | Yes | Yes | Yes | Yes | Yes | No | Yes | No | Yes | **15/20 (75%)** |
| Park et al. 2022 | Yes | Yes | Yes | No | Yes | No | No | Yes | Yes | No | Yes | No | No | Yes | No | No | Yes | Yes | No | Yes | **10/20 (50%)** |
| Payton et al. 2022 | Yes | Yes | Yes | Yes | Yes | No | No | Yes | Yes | Yes | No | No | Yes | Yes | Yes | Yes | Yes | Yes | No | Yes | **15/20 (70%)** |
| Pearson et al. 2019 | No | Yes | No | No | Yes | No | No | No | No | No | No | No | No | Yes | No | No | No | Yes | No | No | **4/20**  **(20%)** |
| Perrin et al. 2021 | Yes | Yes | Yes | Yes | Yes | Yes | No | Yes | Yes | Yes | Yes | Yes | Yes | Yes | Yes | Yes | Yes | Yes | No | Yes | **19/20 (90%)** |
| Pratt et al 2022 | Yes | Yes | Yes | No | Yes | No | No | Yes | No | Yes | No | Yes | Yes | Yes | No | No | Yes | Yes | No | Yes | **12/20 (55%)** |
| Purkey et al. 2019 | Yes | Yes | Yes | Yes | Yes | Yes | No | Yes | Yes | No | No | Yes | Yes | Yes | Yes | No | Yes | Yes | No | Yes | **17/20 (85%)** |
| Ramirez et al. 2011 | No | Yes | Yes | No | Yes | No | No | Yes | Yes | Yes | Yes | Yes | Yes | Yes | No | No | Yes | Yes | No | Yes | **13/20 (65%)** |
| Roura et al. 2015 | No | Yes | Yes | No | Yes | No | No | Yes | Yes | No | Yes | Yes | No | Yes | No | No | Yes | Yes | No | Yes | **11/20 (55%)** |
| Scorgie et al. 2017 | No | Yes | Yes | No | Yes | No | No | Yes | Yes | Yes | Yes | Yes | Yes | Yes | No | Yes | Yes | Yes | No | Yes | **14/20 (70%)** |
| Shabu et al. 2022 | Yes | Yes | Yes | Yes | Yes | Yes | No | Yes | Yes | No | Yes | No | Yes | Yes | No | No | Yes | No | No | No | **12/20 (60%)** |
| Spurling et al. 2017 | Yes | Yes | Yes | Yes | Yes | No | No | Yes | Yes | No | Yes | Yes | Yes | Yes | Yes | No | Yes | Yes | No | No | **14/20 (70%)** |
| Stone et al. 2020 | Yes | Yes | Yes | Yes | Yes | Yes | No | Yes | No | Yes | No | No | No | Yes | Yes | Yes | Yes | Yes | No | No | **13/20 (65%)** |
| Tamlyn et al. 2023 | Yes | Yes | Yes | Yes | Yes | No | No | Yes | Yes | Yes | Yes | No | Yes | Yes | Yes | Yes | Yes | Yes | No | Yes | **16/20 (80%)** |
| Turin et al. 2021 | No | Yes | Yes | Yes | Yes | No | No | Yes | Yes | Yes | Yes | No | No | Yes | No | No | No | Yes | No | Yes | **11/20 (55%)** |
| Valerio et al. 2016 | No | Yes | No | Yes | Yes | No | No | No | Yes | Yes | Yes | No | Yes | Yes | No | No | Yes | Yes | No | Yes | **11/20 (55%)** |
| Varese et al. 2023 | Yes | Yes | Yes | No | Yes | No | No | Yes | Yes | Yes | Yes | Yes | Yes | Yes | No | No | Yes | Yes | No | Yes | **14/20 (70%)** |
| Wapau et al 2022 | Yes | Yes | Yes | No | Yes | Yes | Yes | Yes | No | No | No | No | Yes | Yes | No | No | Yes | Yes | No | Yes | **12/20 (60%)** |
| Watson et al. 2013 | No | Yes | Yes | Yes | Yes | No | No | Yes | Yes | Yes | Yes | Yes | No | Yes | Yes | Yes | No | Yes | No | Yes | **15/20 (75%)** |
| Wong et al. 2015 | No | Yes | Yes | No | Yes | No | No | Yes | Yes | Yes | Yes | Yes | Yes | No | Yes | No | No | Yes | No | Yes | **10/20 (50%)** |
| Yan et al. 2020 | No | Yes | Yes | Yes | Yes | No | No | Yes | Yes | Yes | Yes | No | Yes | Yes | Yes | No | Yes | Yes | No | Yes | **14/20 (70%)** |
| Zeh et al. 2018 | Yes | Yes | Yes | Yes | Yes | No | No | Yes | Yes | Yes | No | No | Yes | Yes | Yes | Yes | Yes | Yes | No | Yes | **15/20 (75%)** |
| Zumeta et al. 2022 | Yes | Yes | Yes | No | No | No | No | Yes | Yes | Yes | No | No | No | Yes | No | No | No | No | No | No | **7/20 (35%)** |
| **TOTAL (No. of studies)** | **27** | **47** | **44** | **24** | **45** | **9** | **4** | **44** | **39** | **36** | **28** | **23** | **30** | **46** | **21** | **15** | **39** | **45** | **0** | **41** |  |
| **TOTAL (%)** | **57** | **100** | **94** | **51** | **96** | **19** | **9** | **94** | **83** | **77** | **60** | **49** | **64** | **98** | **45** | **32** | **83** | **96** | **0** | **87** |  |

# Reference list of Included Studies

1. Alotaibi A, Saleh W, Abdulbaqi A, Alosaimi M. Health research priority agenda for Ministry Of Health, Kingdom of Saudi Arabia from 2020 to 2025. Journal of Epidemiology and Global Health. 2022; 12:413-29.

2. Asnaani A, Sanchez-Birkhead A, Kaur K, Mukundente V, Napia E, Tavake-Pasi F, et al. Utilizing community partnerships to devise a framework for cultural adaptations to evidence-based mental health practice in diverse communities. Cognitive and Behavioral Practice. 2022; 29:831-45.

3. Borthwick J, Evertsz N, Pratt B. How should communities be meaningfully engaged (if at all) when setting priorities for biomedical research? Perspectives from the biomedical research community. BMC Medical Ethics. 2023; 24:6.

4. Bowers B, Jacobson N, Krupp A. Can lay community advisors improve the clarity of research participant recruitment materials and increase the likelihood of participation? Research in nursing & health. 2017; 40:63-9.

5. Bowring F, Welch J, Woodward C, Lo C, Lawton M, Sulzer P, et al. Exploration of whether socioeconomic factors affect the results of priority setting partnerships: updating the top 10 research priorities for the management of Parkinson’s in an international setting. BMJ open. 2022; 12:e049530.

6. Bryan S, Goldsmith LJ, Suryaprakash N, Sawatzky R, Mulldoon M, Le Mercier M, et al. A research agenda to improve patients’ experience of knee replacement surgery: a patient-oriented modified Delphi study of patients of South Asian origin in British Columbia. Canadian Medical Association Open Access Journal. 2020; 8:E226-E33.

7. Bryant K, Pro G, Rojo M, Patel J, Haynes T, McElfish P, et al. Identifying community needs of the Hispanic faith community to develop a research agenda. Public Health Nursing. 2022; 39:33-9.

8. Cartwright C, Rahman A, Islam S, Lockyer B, Roper E, Worcester M, et al. People powered research: what do communities identify as important for happy and healthy children and young people? A multi-disciplinary community research priority setting exercise in the City of Bradford, United Kingdom (UK). International Journal for Equity in Health. 2023; 22:71.

9. Chlebowski C, Hurwich‐Reiss E, Wright B, Brookman‐Frazee L. Using stakeholder perspectives to guide systematic adaptation of an autism mental health intervention for Latinx families: A qualitative study. Journal of Community Psychology. 2020; 48:1194-214.

10. Dirks LG, Shaw JL, Hiratsuka VY, Beans JA, Kelly JJ, Dillard DA. Perspectives on communication and engagement with regard to collecting biospecimens and family health histories for cancer research in a rural Alaska Native community. Journal of Community Genetics. 2019; 10:435-46.

11. Erves JC, Mayo-Gamble TL, Malin-Fair A, Boyer A, Joosten Y, Vaughn YC, et al. Needs, priorities, and recommendations for engaging underrepresented populations in clinical research: a community perspective. Journal of community health. 2017; 42:472-80.

12. Finer S, Robb P, Cowan K, Daly A, Shah K, Farmer A. Setting the top 10 research priorities to improve the health of people with Type 2 diabetes: a Diabetes UK–James Lind Alliance Priority Setting Partnership. Diabetic Medicine. 2018; 35:862-70.

13. Franck LS, McLemore MR, Cooper N, De Castro B, Gordon AY, Williams S, et al. A novel method for involving women of color at high risk for preterm birth in research priority setting. JoVE (Journal of Visualized Experiments). 2018:e56220.

14. Goold SD, Myers CD, Szymecko L, Cunningham Collins C, Martinez S, Ledón C, et al. Priorities for patient‐centered outcomes research: the views of minority and underserved communities. Health services research. 2017; 52:599-615.

15. Goold SD, Myers CD, Danis M, Abelson J, Barnett S, Calhoun K, et al. Members of minority and underserved communities set priorities for health research. The Milbank Quarterly. 2018; 96:675-705.

16. Green M, Anderson K, Griffiths K, Garvey G, Cunningham J. Understanding Indigenous Australians’ experiences of cancer care: Stakeholders’ views on what to measure and how to measure it. BMC health services research. 2018; 18:1-13.

17. Iqbal H, West J, McEachan RRC, Haith‐Cooper M. Exploring the obesity concerns of British Pakistani women living in deprived inner‐city areas: A qualitative study. Health Expectations. 2022; 25:1821-31.

18. Kreuter MW, Kegler MC, Joseph KT, Redwood YA, Hooker M. The impact of implementing selected CBPR strategies to address disparities in urban Atlanta: A retrospective case study. Health education research. 2012; 27:729-41.

19. Maar MA, Seymour A, Sanderson B, Boesch L. Reaching agreement for an Aboriginal e-health research agenda: the Aboriginal telehealth knowledge circle consensus method. Rural and remote health. 2010; 10:100-12.

20. MacFarlane A, Galvin R, O’Sullivan M, McInerney C, Meagher E, Burke D, et al. Participatory methods for research prioritization in primary care: an analysis of the World Café approach in Ireland and the USA. Family practice. 2017; 34:278-84.

21. Manikam L, Shah R, Reed K, Santini G, Lakhanpaul M. Using a co‐production prioritization exercise involving South Asian children, young people and their families to identify health priorities requiring further research and public awareness. Health Expectations. 2017; 20:852-61.

22. McMorrow S, Saksena J. Voices and views of Congolese refugee women: A qualitative exploration to inform health promotion and reduce inequities. Health Education & Behavior. 2017; 44:769-80.

23. Mohamed AA, Lantz K, Ahmed YA, Osman A, Nur MA, Nur O, et al. An assessment of health priorities among a community sample of Somali adults. Journal of immigrant and minority health. 2022:1-6.

24. Montesanti S, Fitzpatrick K, Fayant B, Pritchard C. Identifying priorities, directions and a vision for Indigenous mental health using a collaborative and consensus-based facilitation approach. BMC health services research. 2022; 22:406.

25. Morris BA, Anderson K, Cunningham J, Garvey G. Identifying research priorities to improve cancer control for Indigenous Australians. Public Health Res Pract. 2017; 27:2741735.

26. Park A, Kennedy A, Kennedy R, Zimmern PE, Malik RD. Identifying Patient-Centered Research Priorities in Overactive Bladder by Crowdsourcing and Targeted Recruitment. Urology Practice. 2022; 9:246-52.

27. Payton C, Kumar GS, Kimball S, Clarke SK, AlMasri I, Karaki FM. A logic model framework for planning an International Refugee Health Research, Evaluation, and Ethics Committee. Health Promotion Practice. 2022; 23:852-60.

28. Pearson CR, Smartlowit-Briggs L, Belcourt A, Bedard-Gilligan M, Kaysen D. Building a tribal–academic partnership to address PTSD, substance misuse, and HIV among American Indian women. Health Promotion Practice. 2019; 20:48-56.

29. Perrin BM, Raspovic A, Williams CM, Twigg SM, Golledge J, Hamilton EJ, et al. Establishing the national top 10 priority research questions to improve diabetes-related foot health and disease: a Delphi study of Australian stakeholders. BMJ Open Diabetes Research and Care. 2021; 9:e002570.

30. Pratt B, Seshadri T, Srinivas PN. Overcoming structural barriers to sharing power with communities in global health research priority-setting: lessons from the Participation for Local Action project in Karnataka, India. Global public health. 2022; 17:3334-52.

31. Purkey E, Htoo SN, Whelan R, Mhote NPP, Davison CM. Creating a locally driven research agenda for the ethnic minorities of Eastern Myanmar. Health Research Policy and Systems. 2019; 17:1-10.

32. Ramirez AG, Chalela P, Gallion KJ, Green LW, Ottoson J. Salud America! Developing a national Latino childhood obesity research agenda. Health Education & Behavior. 2011; 38:251-60.

33. Roura M, Bisoffi F, Navaza B, Pool R. " Carrying Ibuprofen in the Bag": Priority Health Concerns of Latin American Migrants in Spain-A Participatory Qualitative Study. PLoS One. 2015; 10:e0136315.

34. Scorgie F, Vearey J, Oliff M, Stadler J, Venables E, Chersich MF, et al. ‘Leaving no one behind’: reflections on the design of community-based HIV prevention for migrants in Johannesburg’s inner-city hostels and informal settlements. BMC public health. 2017; 17:1-12.

35. Shabu T, Espinoza AM, Manning S, Cardarelli R, Fulda KG. Patient research interest differences by gender and race/ethnicity: a North Texas primary care practice-based research network (NorTex) study. The Journal of the American Board of Family Medicine. 2022; 35:225-34.

36. Spurling GK, Bond CJ, Schluter PJ, Kirk CI, Askew DA. ‘I’m not sure it paints an honest picture of where my health’s at’–identifying community health and research priorities based on health assessments within an Aboriginal and Torres Strait Islander community: a qualitative study. Australian Journal of Primary Health. 2018; 23:549-53.

37. Torres Stone RA, Cardemil EV, Keefe K, Bik P, Dyer Z, Clark KE. A community mental health needs assessment of a racially and ethnically diverse population in New England: Narratives from community stakeholders. Community Mental Health Journal. 2020; 56:947-58.

38. Tamlyn AL, Tjilos M, Bosch NA, Barnett KG, Perkins RB, Walkey A, et al. At the intersection of trust and mistrust: A qualitative analysis of motivators and barriers to research participation at a safety‐net hospital. Health Expectations. 2023; 26:1118-26.

39. Turin TC, Haque S, Chowdhury N, Yeasmin F, Ferdous M, Rashid R, et al. Community-Driven prioritization of primary health care access issues by Bangladeshi-Canadians to guide program of research and practice. Family & Community Health. 2021; 44:292-8.

40. Valerio MA, Rodriguez N, Winkler P, Lopez J, Dennison M, Liang Y, et al. Comparing two sampling methods to engage hard-to-reach communities in research priority setting. BMC medical research methodology. 2016; 16:1-11.

41. Varese F, White C, Longden E, Charalambous C, Meehan K, Partington I, et al. Top 10 priorities for sexual violence and abuse research: findings of the James Lind alliance sexual violence priority setting partnership. BMJ open. 2023; 13:e062961.

42. Wapau H, Kris E, Roeder L, McDonald M. Community-driven health research in the Torres Strait. Australian Journal of Primary Health. 2022; 28:289-95.

43. Watson M-R, Kaltman S, Townsend TG, Goode T, Campoli M. A collaborative mental health research agenda in a community of poor and underserved Latinos. Journal of health care for the poor and underserved. 2013; 24:671-87.

44. Wong A, Peiris-John R, Sobrun-Maharaj A, Ameratunga S. Priorities and approaches to investigating Asian youth health: perspectives of young Asian New Zealanders. Journal of primary health care. 2015; 7:282-90.

45. Yan A, Millon-Underwood S, Walker A, Patten C, Nevels D, Dookeran K, et al. Engaging young African American women breast cancer survivors: a novel storytelling approach to identify patient‐centred research priorities. Health Expectations. 2020; 23:473-82.

46. Zeh P, Cannaby A-M, Sandhu HK, Warwick J, Sturt JA. A cross-sectional survey of general practice health workers’ perceptions of their provision of culturally competent services to ethnic minority people with diabetes. Primary Care Diabetes. 2018; 12:501-9.

47. Zumeta L, Bobowik M, Basabe N, Wlodarczyk A. Participation in multicultural awareness-raising community actions: Positive effects on well-being and group efficacy. Cultur Divers Ethnic Minor Psychol. 2022; 28:413-26.
